# The ubiquitin ligase HECTD1 promotes retinoic acid signaling required for development of the aortic arch

**DOI:** 10.1242/dmm.036491

**Published:** 2019-01-11

**Authors:** Kelsey F. Sugrue, Anjali A. Sarkar, Linda Leatherbury, Irene E. Zohn

**Affiliations:** 1 Institute for Biomedical Sciences, The George Washington University, Washington, DC 20037, USA; 2 Center for Genetic Medicine Research, Children's National Health System, Washington, DC 20010, USA; 3 Children's National Heart Institute, Children's National Health System, George Washington University School of Medicine, Washington, DC 20010, USA

**Keywords:** Congenital heart defects, *Hectd1*, Retinoic acid, Vitamin A deficiency, Aortic arch development

## Abstract

The development of the aortic arch is a complex process that involves remodeling of the bilaterally symmetrical pharyngeal arch arteries (PAAs) into the mature asymmetric aortic arch. Retinoic acid signaling is a key regulator of this process by directing patterning of the second heart field (SHF), formation of the caudal PAAs and subsequent remodeling of the PAAs to form the aortic arch. Here, we identify the HECTD1 ubiquitin ligase as a novel modulator of retinoic acid signaling during this process. *Hectd1^opm/opm^* homozygous mutant embryos show a spectrum of aortic arch abnormalities that occur following loss of 4th PAAs and increased SHF marker expression. This sequence of defects is similar to phenotypes observed in mutant mouse models with reduced retinoic acid signaling. Importantly, HECTD1 binds to and influences ubiquitination of the retinoic acid receptor, alpha (RARA). Furthermore, reduced activation of a retinoic acid response element (RARE) reporter is detected in *Hectd1* mutant cells and embryos. Interestingly, *Hectd1^opm/+^* heterozygous embryos exhibit reduced retinoic acid signaling, along with intermediate increased expression of SHF markers; however, heterozygotes show normal development of the aortic arch. Decreasing retinoic acid synthesis by reducing *Raldh2* (also known as *Aldh1a2*) gene dosage in *Hectd1^opm/+^* heterozygous embryos reveals a genetic interaction. Double heterozygous embryos show hypoplasia of the 4th PAA and increased incidence of a benign aortic arch variant, in which the transverse arch between the brachiocephalic and left common carotid arteries is shortened. Together, our data establish that HECTD1 is a novel regulator of retinoic acid signaling required for proper aortic arch development.

## Abstract (precis)

**Editor's choice:** The HECTD1 ubiquitin ligase is a novel modulator of retinoic acid signaling during aortic arch development and provides a model for complex interactions underlying variations in aortic arch development.

## INTRODUCTION

Congenital heart defects (CHDs) are the most common structural birth defects in humans, occurring in ∼1% of live births, with abnormalities of the outflow tract and the aortic arch accounting for 20-30% of all CHDs (Bruneau, 2008; Creazzo et al., 1998; Fahed et al., 2013; Lloyd-Jones et al., 2009). The aortic arch is a key conduit for blood flow from the heart to the systemic circulation. The aortic arch consists of the aorta, the brachiocephalic artery that divides into the right subclavian and right common carotid arteries, the left common carotid artery, the left subclavian artery and the ductus arteriosus/arterial duct. Some of the most common clinically significant abnormalities of the aortic arch include persistent truncus arteriosus, right-sided aortic arch, coarctation of the aorta, interrupted aortic arch and patent ductus arteriosus (Kau et al., 2007; van der Linde et al., 2011). There are also a number of normal anatomical aortic arch variants found in the human population, with the most common being a common origin for the brachiocephalic artery and left common carotid arteries. This variant occurs in 10-20% of the population and is sometimes called a ‘bovine arch’ because of structural similarities to aortic arch of cattle; however, the variant in humans is not a true bovine arch, since in cattle there is a single brachiocephalic trunk origin for the carotid and subclavian arteries (Moorehead et al., 2016; Shaw et al., 2003; Spacek and Veselka, 2012; Stewart, 1964). The underlying cause of these normal anatomical variants remains unknown.

The basic branching organization of the mature aortic arch is established by 8 weeks of gestation in the human embryo and by embryonic day (E) 14.5 in the mouse. Development of the aortic arch is complex and involves significant remodeling of the bilaterally symmetrical embryonic pharyngeal arch arteries (PAAs). Paired 1st through 6th PAAs successively form and undergo asymmetric remodeling to form different aspects of the vasculature and aortic arch (Hiruma et al., 2002). The 1st and 2nd PAAs transiently support blood flow in the embryo and then remodel to form portions of the arteries of the head and neck, whereas the 3rd, 4th and 6th PAAs remodel to contribute to the aortic arch network (Hiruma et al., 2002). The 5th PAAs do not contribute to the mature aortic arch in mammals, and whether the 5th PAA forms at all or only forms transiently then rapidly regresses remains debatable (Bamforth et al., 2013; de Ruiter et al., 1989; Geyer and Weninger, 2012). The left and right 3rd PAAs contribute to the respective carotid arteries, whereas the left 4th PAA contributes to the aortic arch and the right 4th PAA to the proximal region of the right subclavian artery (Hiruma et al., 2002). Whereas the right 6th PAA regresses, the left 6th PAA contributes to the proximal pulmonary arteries and the ductus arteriosus, which connects the aortic arch and pulmonary trunk during fetal development, then closes at birth (Hiruma et al., 2002; Kau et al., 2007; Kaufman and Bard, 1999; Stewart, 1964).

Formation of the PAAs and remodeling into the aortic arch involves complex interactions between multiple cell types. The endothelial cells that line the 3rd through 6th PAAs are derived from a subset of the splanchnic mesoderm known as the second heart field (SHF) (Verzi et al., 2005; Wang et al., 2017). SHF cells migrate to the developing pharyngeal mesenchyme, where they organize into an endothelial plexus that coalesces to form the endothelium of these PAAs (Li et al., 2012; Wang et al., 2017). Neural crest cells migrate from the dorsal neural tube to the pharyngeal arches and contribute to the smooth muscles of the 3rd, 4th and 6th PAAs as well as the pharyngeal mesenchyme (Keyte and Hutson, 2012). Although not required for formation of the caudal PAAs, the cardiac neural crest plays essential roles in remodeling of these PAAs to form the mature aortic arch (Bockman et al., 1987; Kirby and Waldo, 1995).

The retinoic acid signaling pathway is a key mediator of the formation and remodeling of the aortic arch. A variety of abnormalities are observed in mutant mouse models affecting retinoic acid signaling and include transposition of the great arteries, interrupted aortic arch, aberrant origin of the right subclavian artery and persistent truncus arteriosus (El Robrini et al., 2016; Ghyselinck et al., 1997; Jiang et al., 2002; Kastner et al., 1994; Lee et al., 1997; Mendelsohn et al., 1994a; Niederreither et al., 2001; Pan and Baker, 2007; Stefanovic and Zaffran, 2017). During development of the heart, retinoic acid signaling is initially required for anterior-posterior patterning of the SHF by restricting the posterior expression limit of key SHF markers, including *Isl1* and *Fgf8* (Ryckebusch et al., 2008; Sirbu et al., 2008). Later, retinoic acid signaling is required to prevent premature differentiation of the SHF, promote migration of SHF cells to the pharyngeal arches and then facilitate the aggregation of SHF cells to form the endothelium of the PAAs (Li et al., 2012). Consequently, both *Raldh2* (also known as *Aldh1a2*) and retinoic acid receptor (RAR) mutant embryos show caudal PAA defects primarily involving the 4th and 6th PAAs (Li et al., 2012; Niederreither et al., 2003; Vermot et al., 2003).

In this study, we identify HECTD1 as a new modulator of retinoic acid signaling during aortic arch development. The *openmind* mouse line (*Hectd1^opm^*) was originally isolated in an N-ethyl-N-nitrosourea (ENU) screen for genes required for embryonic development and harbors a missense mutation in the *Hectd1* gene (Kasarskis et al., 1998; Zohn et al., 2007). Our previous work characterized neural tube closure and placental defects in homozygous *Hectd1^opm/opm^* mutants (Sarkar et al., 2014, 2016; Sarkar and Zohn, 2012; Zohn et al., 2007). Recently, another ENU-induced allele of *Hectd1* (*Hectd1^b2b327Clo^*) was identified in a CHD screen in mouse, with reported defects including double outlet right ventricle, aortic atresia, hypoplastic aortic arch and ventricular septal defects (Li et al., 2015b); however, the range of aortic arch abnormalities was not characterized, nor were the underlying developmental mechanisms investigated. In this study, we characterize aortic arch malformations in *Hectd1^opm^* mutant embryos and demonstrate that defects are preceded by abnormalities in PAA and SHF development. Furthermore, we show that HECTD1 binds to the retinoic acid receptor, alpha (RARA) and that retinoic acid signaling is reduced in *Hectd1* mutant embryos. Our data demonstrate that loss of *Hectd1* gene function results in reduced retinoic acid signaling in the embryo, leading to aberrant patterning of the SHF and subsequent PAA and aortic arch abnormalities.

## RESULTS

### Altered aortic arch anatomy in *Hectd1^opm^* mutants

**Fig. 1.**
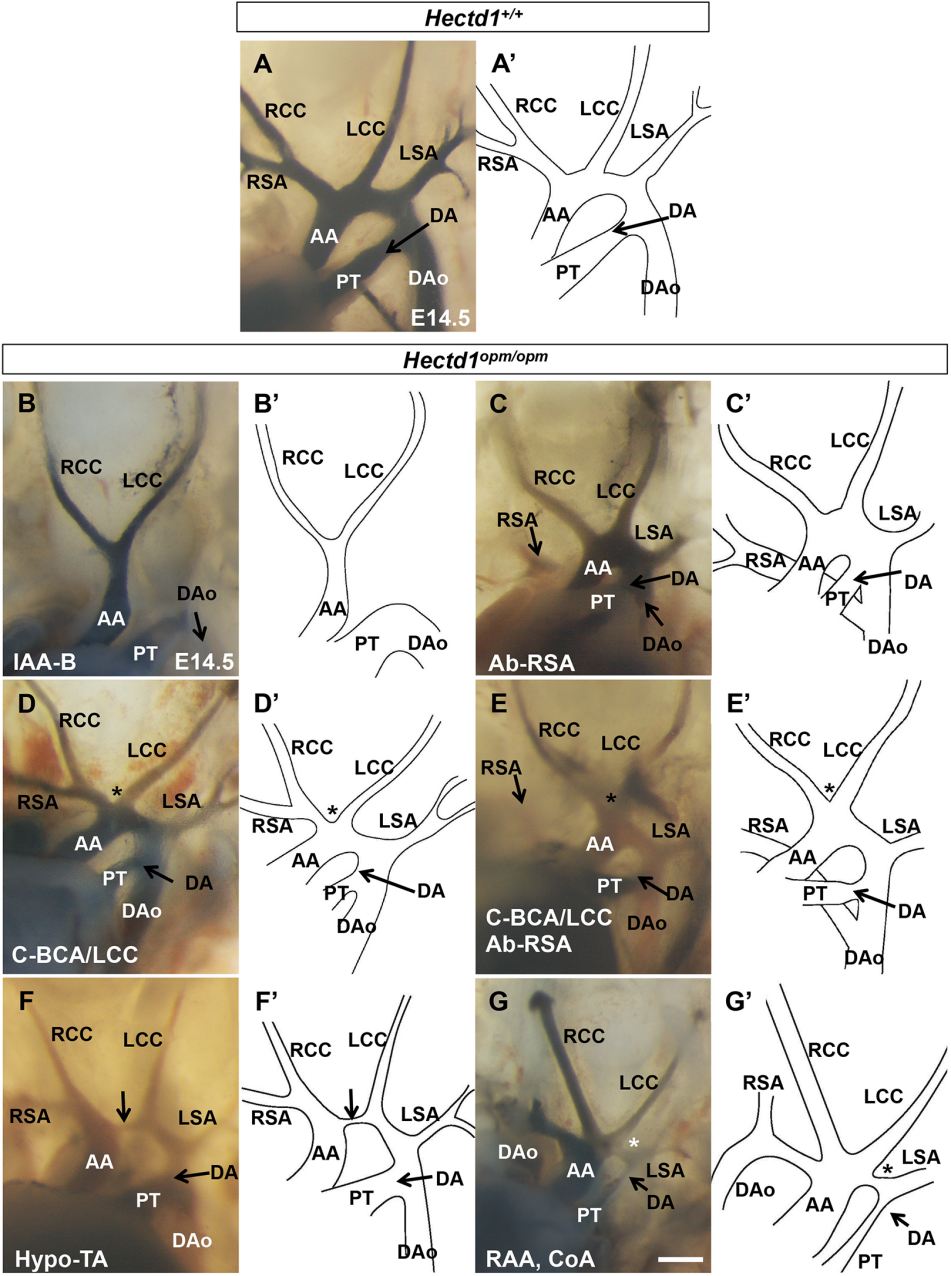
**Abnormalities of the aortic arch in *Hectd1^opm/opm^* mutant embryos.** (A) Intracardiac ink injections of E14.5 wild-type embryos (*n*=10), highlighting the normal mature artery architecture. A′-G′ show tracings of ink-labeled arteries in A-G. (B-G) 90% of the *Hectd1^opm/opm^* mutants analyzed demonstrated aortic arch abnormalities (*n*=9/10; Table 1) that included interrupted aortic arch type B (*n*=5; IAA-B) (B), aberrant origin of the right subclavian artery (*n*=4; Ab-RSA) (C), common origin of the brachiocephalic and left common carotid arteries (*n*=3; C-BCA/LCC, black asterisks) (D) or both C-BCA/LCC and Ab-RSA (*n*=2) (E). Other variants such as hypoplasia of the transverse arch (*n*=2; Hypo-TA, arrow) (F) and right-sided aortic arch with coarctation of the aorta (*n*=2; RAA, CoA, white asterisk) (G) were also observed. AA, ascending aorta; DA, ductus arteriosus; DAo, descending aorta; LCC, left common carotid; LSA, left subclavian artery; PT, pulmonary trunk; RCC, right common carotid; RSA, right subclavian artery. Scale bar: 250 µm.

A forward genetic screen in mouse used to identify new candidate genes for CHDs isolated a mutant allele of *Hectd1* (*Hectd1^b2b327Clo^*), with aortic arch abnormalities including hypoplastic aortic arch and aortic atresia (Li et al., 2015b); however, the full range of aortic arch malformations was not characterized. To determine whether the *Hectd1^opm^* mutant mouse line also shows similar abnormalities, the aortic arch was visualized in wild type and *Hectd1^opm/opm^* homozygous mutants by intracardiac India ink injection at E14.5. This developmental stage was chosen because *Hectd1^opm/opm^* homozygous mutants die shortly after E14.5 (Sarkar et al., 2014). We observed that 90% (*n*=10) of the wild-type embryos and 95% (*n*=20) of the *Hectd1^opm/+^* heterozygous embryos examined displayed normal aortic arch architecture (Table 1, Fig. 1A). One wild-type and one *Hectd1^opm/+^* heterozygous embryo exhibited a benign aortic arch variation, where the transverse arch is shortened between the brachiocephalic and left common carotid arteries. In contrast, 90% (*n*=18/20) of the *Hectd1^opm/opm^* mutants analyzed showed abnormalities of the aortic arch (Table 1). The most common anomalies were interrupted aortic arch type B (*n*=5, Fig. 1B), aberrant right subclavian artery (*n*=4, Fig. 1C), and a common origin between the brachiocephalic and left common carotid arteries (*n*=3, Fig. 1D). Additionally, two *Hectd1^opm/opm^* mutant embryos had a combined phenotype of common origin between the brachiocephalic and left common carotid arteries, and aberrant right subclavian artery (Fig. 1E). Other abnormalities observed included a hypoplastic transverse arch (*n*=2, Fig. 1F) and right-sided aortic arch with coarctation of the aorta (*n*=2, Fig. 1G). Thus, mutation of *Hectd1* results in a spectrum of aortic arch abnormalities involving the aortic arch and right subclavian artery.

**Table 1.**
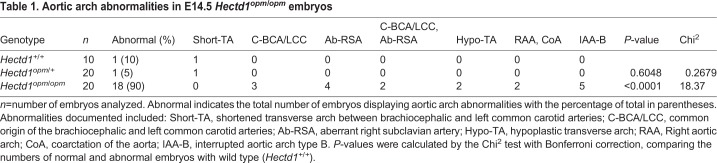
**Aortic arch abnormalities in E14.5 *Hectd1^opm/opm^* embryos**

### Loss of 4th PAAs in *Hectd1^opm^* mutants

**Fig. 2.**
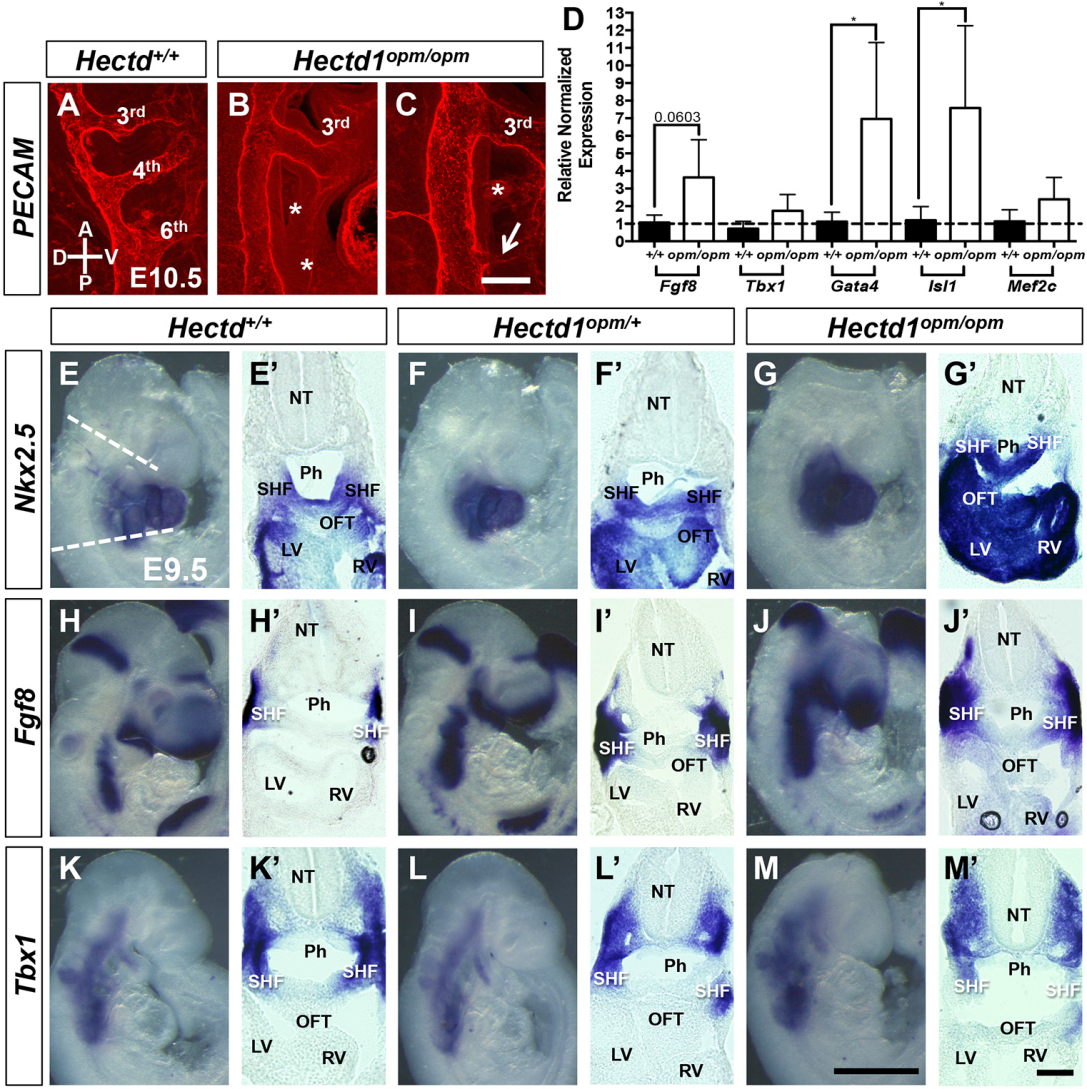
**4th PAA and SHF defects in *Hectd1^opm/opm^* mutant embryos.** (A-C) Right-sided view of E10.5 (32- to 36-somite) embryos stained for PECAM1 to visualize the endothelium of the PAAs. (A) All but one wild-type embryos (*n*=9/10) have well-formed 3rd, 4th and 6th PAAs. (B,C) The 4th PAA was absent in the majority (*n*=8/10) of *Hectd1^opm/opm^* mutant embryos, with bilateral loss of the 4th PAA in 6/10 embryos and two embryos with loss of only the left or right PAA (B,C, asterisks, Table 2). The 6th PAA was also affected in all embryos, with phenotypes consisting of bilateral loss in six embryos (B, asterisks), bilateral hypoplasia in two embryos, left side loss in one embryo, and loss of the left and hypoplasia on the right in another (C, arrow). (D) Quantitation of SHF marker expression by qPCR for *Fgf8*, *Tbx1*, *Gata4*, *Isl1* and *Mef2c* was analyzed in dissected pharyngeal explants (*n*=5; dissected along the dashed lines in E). Data are shown as fold expression relative to wild type and significance was measured by unpaired two-tailed Student's *t*-test. All markers were elevated in *Hectd1^opm/opm^* mutant tissue, with *Gata4* and *Isl1* being significantly increased (*P<0.05) and *Fgf8* almost statistically significantly increased (*P*=0.06). Error bars represent s.e.m. (E-M) Whole-mount *in situ* hybridization to visualize *Nkx2.5* in the cardiac mesoderm (E-G) or *Fgf8* (H-J) and *Tbx1* (K-M) in the SHF of E9.5 wild-type (*Hectd1^+/+^*; E,H,K), *Hectd1^opm/+^* (F,I,L) and *Hectd1^opm/opm^* (G,J,M) embryos. (E′-M′) Expression of markers of the SHF was further visualized in sectioned embryos. Increased expression of *Nkx2.5* and *Fgf8* was observed in 3/5 (60%) and 3/5 (60%) of *Hectd1^opm/+^* heterozygous mutants, and 3/3 (100%) and 5/5 (100%) of *Hectd1^opm/opm^* homozygous mutants, respectively, compared with wild type (*Nkx2.5 n*=5; *Fgf8 n*=5). *Tbx1* expression was similar across genotypes in *Hectd1^opm/opm^* mutants (wild type, *n*=5; *Hectd1^opm/+^*, *n*=4; *Hectd1^opm/opm^*, *n*=3). Scale bars: 50 µm (C,M′) and 250 µm (M). A, anterior; D, dorsal; LV, left ventricle; NT, neural tube; OFT, outflow track; P, posterior; Ph, pharynx; RV, right ventricle; SHF, second heart field; V, ventral.

The aortic arch and the right subclavian artery are derived from the left and right 4th PAAs, respectively, and loss of the 4th PAA can cause the types of aortic arch abnormalities observed in *Hectd1^opm/opm^* mutant embryos (Hiruma et al., 2002; Stewart, 1964). Thus, to determine the developmental origins of aortic arch anomalies in *Hectd1^opm/opm^* mutant embryos, formation of the 4th PAA was analyzed in E10.5 embryos (32- to 36-somite stage) by whole-mount immunohistochemistry using an anti-PECAM1 antibody to visualize the endothelial cells of the PAAs. While the vast majority (90%; *n*=9/10) of wild-type embryos had well-formed 4th PAAs (Fig. 2A, Table 2), a single wild-type embryo showed bilateral absence of the 4th PAA (Table 2), demonstrating some background phenotypes in our mouse strain. Examination of PAA organization in *Hectd1^opm/opm^* mutants revealed that 80% (8/10) of mutant embryos present with abnormal 4th PAAs (Table 2). The most common 4th PAA phenotype observed was bilateral absence of the 4th PAA (*n*=6), whereas one *Hectd1^opm/opm^* mutant presented with an absent left 4th PAA and another with an absent right 4th PAA (Fig. 2B,C, Table 2). These PAA phenotypes are consistent with the spectrum of aortic arch abnormalities involving the aortic arch and right subclavian artery found in E14.5 *Hectd1^opm/opm^* mutants.

**Table 2.**
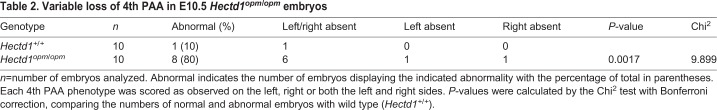
**Variable loss of 4th PAA in E10.5 *Hectd1^opm/opm^* embryos**

### Altered SHF patterning in *Hectd1^opm^* mutant embryos

Proper formation of the PAAs requires contributions from different cell populations, including the SHF and cardiac neural crest. Previous work from our laboratory demonstrated that neural crest cell formation and migration to the heart was unaffected in *Hectd1^opm/opm^* mutants (Zohn et al., 2007). However, failure of 4th PAA formation and subsequent aortic arch abnormalities can arise from alterations in SHF formation, patterning and/or differentiation (Hiruma et al., 2002; Li et al., 2012; Wang et al., 2017). *Nkx2.5* is one of the earliest known markers of the cardiac lineage and is expressed in both the primary and second heart fields (Lyons et al., 1995). *In situ* hybridization revealed a gene-dosage-dependent increase in expression of *Nkx2.5* in *Hectd1^opm/+^* heterozygous and *Hectd1^opm/opm^* homozygous mutant embryos (Fig. 2E-G). Increased *Nkx2.5* expression in the SHF was verified in sectioned embryos (Fig. 2E′-G′). A similar gene-dosage-dependent increase in expression was seen for *Fgf8* in the SHF of *Hectd1^opm/+^* and *Hectd1^opm/opm^* mutant embryos, in both whole-mount and sectioned embryos (Fig. 2H-J). Increase in *Fgf8* expression was verified by quantitative PCR (qPCR) of the pharyngeal region of *Hectd1^opm/opm^* mutant embryos (*n*=5; *P*=0.06; Fig. 2D). However, expression of the SHF marker *Tbx1* was not obviously different between genotypes, nor was expression significantly increased in the pharyngeal region by qPCR (Fig. 2D,K-M). Expression of *Isl1* and *Gata4*, additional SHF markers, was increased in the pharyngeal region of *Hectd1^opm/opm^* mutant embryos as measured by qPCR (*n*=5; *P*<0.05; Fig. 2D), whereas expression of *Mef2c* was not significantly elevated. Together, these results demonstrate that altered patterning of the SHF precedes PAA and aortic arch abnormalities in *Hectd1^opm/opm^* mutants.

### HECTD1 interacts with RARA

**Fig. 3.**
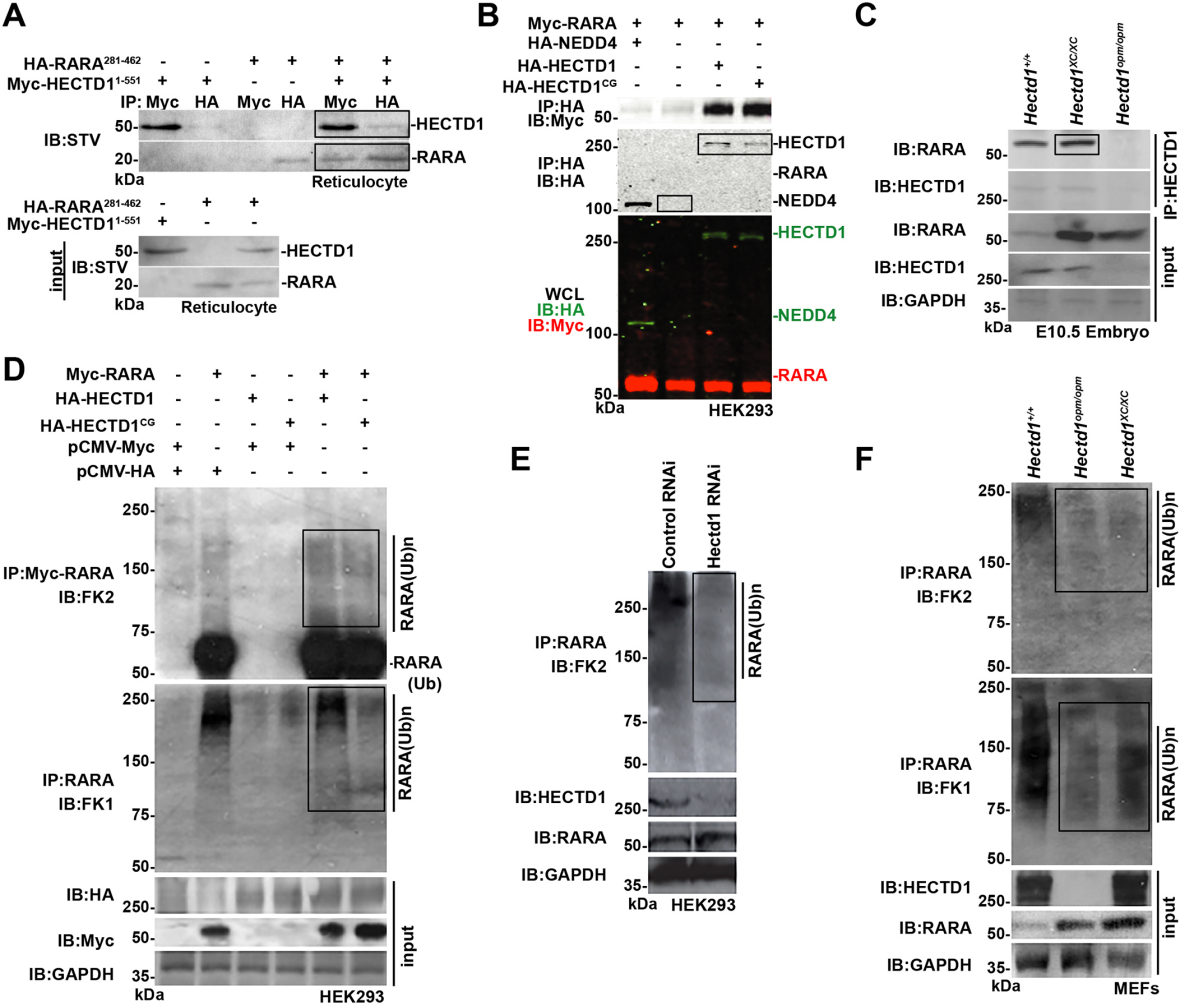
**HECTD1 binds to RARA and influences its ubiquitination.** (A) HA-RARA(281-462), binds to Myc-HECTD1(1-551) expressed in rabbit reticulocyte lysates. Proteins were immunoprecipitated (IP) as indicated followed by immunoblotting (IB) with streptavidin (STV). (B) RARA binds to HA-HECTD1 and ligase-deficient HA-HECTD1^C2579G^ (HA-HECTD11^CG^), but not to HA-NEDD4, another HECT domain-containing E3 ligase, in HEK293T cells. WCL, whole-cell lysate. (C) RARA binds HECTD1 *in vivo* in embryo lysates prepared from E10.5 wild-type and *Hectd1^XC/XC^* mutant embryos. The epitope recognized by the HECTD1 antibody used for immunoprecipitation is not present in *Hectd1^opm/opm^* mutant embryos, thus the failure of HECTD1^opm^ to pull down RARA serves as a negative control. (D) HECTD1 influences ubiquitination of RARA. HEK293T cells were transfected with Myc-RARA and either HA-HECTD1 or ligase-deficient HECTD1^C2579G^. RARA was immunoprecipitated followed by immunoblotting with the FK2 antibody to detect poly- and monoubiquitinated RARA, or FK1 antibody to detect polyubiquitinated RARA. FK2 immunostaining indicates a slight increase in ubiquitinated RARA levels with expression of either wild-type or ligase-defective HECTD1, whereas FK1 immunostaining shows reduced ubiquitinated RARA in HA-HECTD1^C2579G^ transfected cells, consistent with the predicted dominant-negative activity of this construct. (E) Knockdown of HECTD1 by siRNA in HEK293T cells results in decreased ubiquitinated RARA. (F) MEFs derived from *Hectd1^opm/opm^* and *Hectd1^XC/XC^* mutant embryos demonstrate reduced ubiquitinated proteins in RARA immunoprecipitates and increased RARA levels in mutant compared with wild-type cells. Key positive signals are highlighted in boxes.

To understand the molecular mechanism underlying abnormal aortic arch development in *Hectd1^opm/opm^* mutants, a yeast two-hybrid screen was performed to identify HECTD1-interacting proteins (Sarkar and Zohn, 2012). A mouse E11.5 library was probed in a yeast two-hybrid screen for clones that bind to the N-terminal 551 amino acids of HECTD1. Out of 4.5 million clones screened, 23 unique clones were identified, including three identical fragments of RARA corresponding to amino acids 281-462 (Fig. S1). The interaction with RARA was interesting, because retinoic acid signaling is essential for patterning of the SHF along with subsequent PAA formation and patterning (Li et al., 2012; Niederreither et al., 2003; Ryckebusch et al., 2008; Sirbu et al., 2008; Vermot et al., 2003). Interaction of HECTD1 and RARA was confirmed in both *in vitro* and *in vivo* binding assays. Myc-HECTD1(1-551) binds to HA-RARA(281-462) *in vitro* when synthesized in the rabbit reticulocyte lysate system (Fig. 3A). Binding of full-length overexpressed RARA and HECTD1 in human embryonic kidney (HEK) 293T cells was only detected under less-stringent binding conditions with the immunoprecipitation buffer (Fig. 3B and data not shown; see Materials and Methods for buffer compositions). Similar results were obtained with ubiquitin-ligase-deficient HECTD1^C2579G^, indicating that the ubiquitin ligase activity of HECTD1 is not required for binding. As a negative control for specificity, RARA failed to bind the HECT-domain ubiquitin ligase NEDD4 (Fig. 3B).

In contrast to exogenous overexpressed proteins, binding of endogenous proteins *in vivo* could be detected under more-stringent immunoprecipitation conditions in radioimmunoprecipitation assay (RIPA) buffer. For these assays, embryos from two different *Hectd1* mutant mouse lines were used (Fig. S1). A gene trap insertion in the *Hectd1^XC^* mouse line results in expression of a truncated HECTD1 protein that retains substrate binding domains, but the C-terminal ubiquitin ligase domain is disrupted by the insertion of a beta-galactosidase (β-gal) cassette (Zohn et al., 2007). The *Hectd1^opm^* mouse line harbors an ENU-induced nonsense mutation that truncates the 2610-amino-acid HECTD1 protein after amino acid 144, deleting the epitope recognized by our anti-HECTD1 antibody (Zohn et al., 2007). HECTD1 can be immunoprecipitated from E10.5 wild-type and *Hectd1^XC/XC^*, but not *Hectd1^opm/opm^*, mutant embryos, providing a negative control for antibody specificity (Fig. 3C). Importantly, RARA co-immunoprecipitates with both wild-type and mutant HECTD1^XC^, demonstrating binding of endogenous proteins *in vivo*, and further supports that an active catalytic HECT domain is not required for the interaction.

### HECTD1 influences ubiquitination of RARA

We next determined whether the interaction of RARA and HECTD1 influences the ubiquitination of RARA. For these experiments, HEK293T cells were transfected with Myc-RARA along with HA-HECTD1 or ligase-deficient HECTD1^C2579G^. Four hours before lysis, cells were treated with an inhibitor [N-acetyl-Leu-Leu-Norleu-al (ALLN)] to prevent proteasomal-mediated degradation of ubiquitinated RARA. Myc-RARA was immunoprecipitated and ubiquitinated RARA was detected by western blotting with the anti-ubiquitin antibodies FK1 and FK2. FK2 detects mono- and polyubiqutinated proteins, whereas FK1 only recognizes polyubiquitinated proteins; however, the relative affinity for the diverse array of polyubiquitinated chains differs between FK1 and FK2 (Emmerich and Cohen, 2015; Fujimuro and Yokosawa, 2005). Western blotting of RARA immunoprecipitates with the FK2 antibody revealed substantial levels of monoubiquitinated RARA and lower amounts of polyubiquitinated RARA in Myc-RARA transfected cells (Fig. 3D). Both poly- and monoubiquitinated RARA were modestly increased with co-transfection of either wild-type or ligase-deficient HECTD1 (Fig. 3D). Similarly, detection of polyubiquitinated RARA species with the antibody revealed higher-molecular-mass ubiquitinated RARA in lysates from Myc-RARA transfected cells, but little, if any, increase in polyubiquitinated RARA with expression of HECTD1 (Fig. 3D). In contrast, expression of the ubiquitin-ligase-deficient HECTD1^C2579G^ resulted in decreased levels of polyubiquitinated RARA, consistent with the predicted dominant-negative activity with mutation of this critical cysteine (Fig. 3D).

The high level of ubiquitinated RARA in the absence of HECTD1 transfection could be due to the expression of endogenous HECTD1 in HEK293T cells (Sarkar and Zohn, 2012) and/or the expression of other ubiquitin ligases that can influence the ubiquitinated state of RARA (Jing et al., 2008; Sato et al., 2012, 2011; Takano et al., 2004; Zhao et al., 2009). To investigate whether endogenous HECTD1 is required for this high basal level of ubiquitinated RARA, HECTD1 was knocked down by expression of an RNA interference (RNAi) construct (Tran et al., 2013). Levels of ubiquitinated RARA, as detected by FK2 western blotting of RARA immunoprecipitates, were decreased compared with levels in HEK cells expressing a control RNAi construct (Fig. 3E). Levels of ubiquitinated RARA were also evaluated in mouse embryonic fibroblasts (MEFs) prepared from wild-type, *Hectd1^opm/opm^* and *Hectd1^XC/XC^* embryos (Fig. 3F). *Hectd1^opm^* represents a null allele, whereas *Hectd1^XC^* produces a protein with a truncated C-terminal HECT domain, disrupting ubiquitin ligase activity of the protein. The levels of ubiquitinated RARA, as detected by FK2 and FK1 antibodies, were decreased in *Hectd1^opm/opm^* and *Hectd1^XC/XC^* mutant MEFs (Fig. 3F). However, the ubiquitinated RARA species detected by the FK1 antibody were less affected in the *Hectd1^XC/XC^* compared with *Hectd1^opm/opm^* MEFs (Fig. 3F). RARA levels were increased in both *Hectd1^opm/opm^* and *Hectd1^XC/XC^* mutant MEFs, indicating that the ubiquitin ligase activity of HECTD1 is required to destabilize RARA (Fig. 3F). Together, these results indicate that HECTD1 influences ubiquitination of RARA.

### Reduced retinoic acid signaling in *Hectd1* mutant cells

**Fig. 4.**
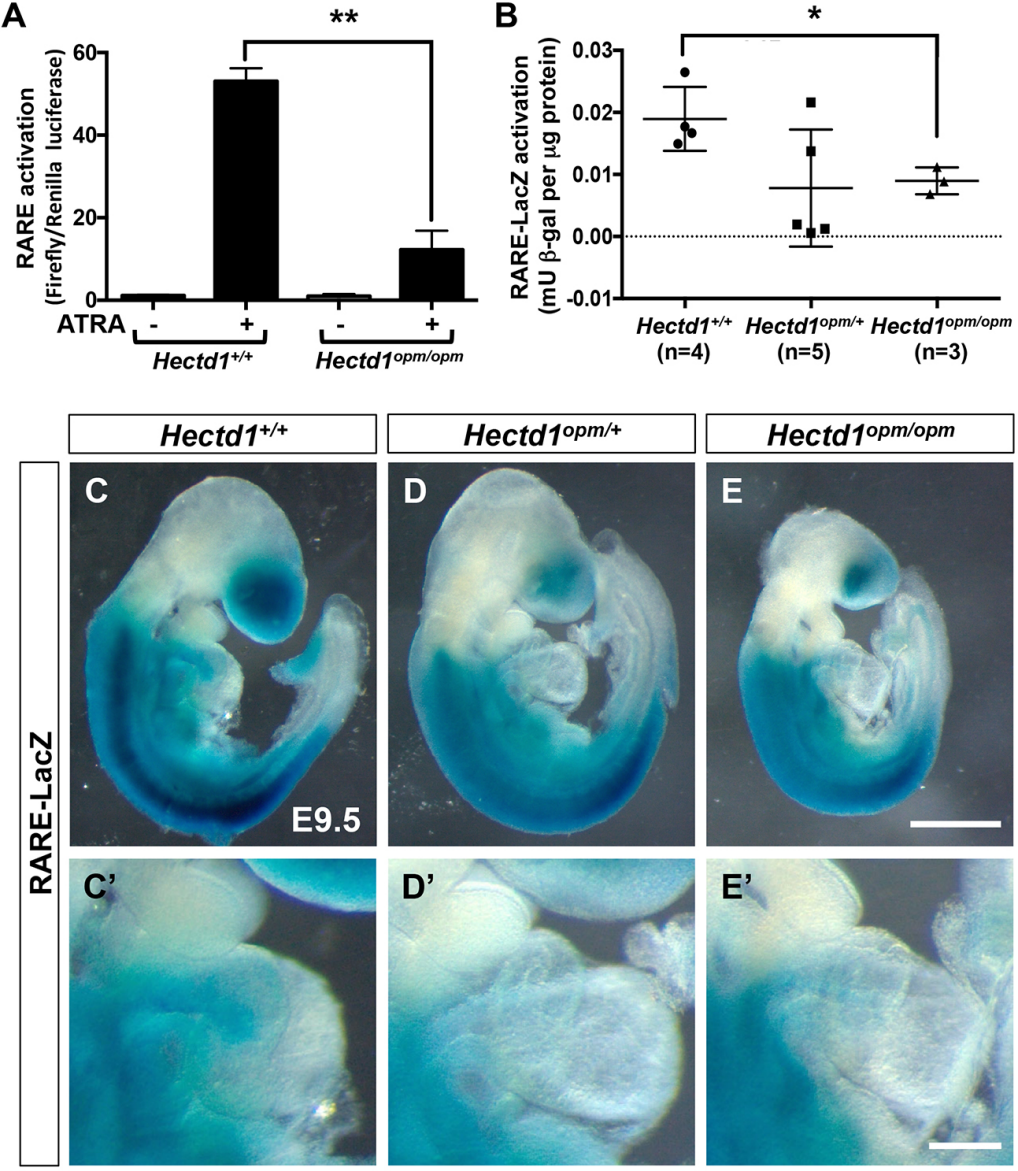
**Activation of a RARE transcriptional reporter is reduced in *Hectd1^opm^* mutants.** (A) Dual luciferase activity was measured in wild-type and *Hectd1^opm/opm^* MEFs transfected with RARE-Firefly luciferase and herpes simplex virus-thymidine kinase-Renilla-luciferase reporter constructs. MEFs were treated with (+) or without (−) 10 µM all-trans retinoic acid (ATRA). ATRA treatment results in significant activation of the RARE reporter in wild-type MEFs, whereas activation is markedly reduced in *Hectd1^opm/opm^* MEFs (**P<0.01). Data shown are representative of experiments performed twice in triplicate. (B) Quantitation of beta-galactosidase (β-gal) activity in whole E9.5 embryos expressing the RARE-LacZ reporter transgene. RARE activation was normalized by dividing β-gal activity (millunits, mU) by total protein content (µg). *Hectd1^opm/opm^* mutants show a significant reduction in RARE activation compared with wild-type littermates (*P<0.05). Although the mean value is reduced, individual data points show that RARE activation in *Hectd1^opm/+^* mutants (*n*=5) is either similar to that in wild type (*n*=4) or *Hectd1^opm/opm^* mutants (*n*=3). (C-E) Whole-mount β-gal staining reveals reduced RARE-LacZ reporter activation in E9.5 *Hectd1^opm/+^* (D; *n*=5) and *Hectd1^opm/opm^* (E; *n*=4) mutant embryos compared with wild-type littermates (C; *n*=4). RARE-LacZ activation is also reduced in the outflow tract region (D′,E′) compared with wild type (C′). Significance was measured by unpaired two-tailed Student's *t*-test and error bars represent s.e.m. Scale bars: 250 µm (E) and 100 µm (E′).

To establish the consequence of the interaction between HECTD1 and RARA on retinoic acid signaling, retinoic acid signaling was evaluated in *Hectd1^opm^* mutant MEFs and embryos. Activation of a RARE-reporter construct was assayed in MEFs from wild-type and *Hectd1^opm/opm^* mutant embryos. Treatment of wild-type MEFs with all-trans retinoic acid (ATRA) resulted in significant activation of transcription from the RARE reporter construct, but ATRA-stimulated transcriptional activation was nearly fourfold less in *Hectd1^opm/opm^* mutant MEFs (*P*<0.01; Fig. 4A). To examine retinoic acid signaling in developing embryos, RARE activation was quantified by measuring β-gal activity in embryos expressing a transgenic RARE-LacZ reporter that consists of three copies of RARE from the RAR-beta (RARB) promoter driving expression of LacZ (Rossant et al., 1991). Quantitation of β-gal activity demonstrates that E9.5 *Hectd1^opm/opm^* mutant embryos show a significant decrease in activation of the RARE-LacZ reporter (*P*<0.05; Fig. 4B). Interestingly, quantification of RARE activation in heterozygous *Hectd1^opm/+^* embryos revealed that individual embryos either showed transcriptional activation similar to what is seen in wild type or reduced activation (Fig. 4B).

This reporter mouse line was also utilized to assess alterations in the spatial pattern of retinoic acid signaling in the embryo. As previously described (Rossant et al., 1991), whole-mount β-gal staining of wild-type embryos at E9.5 reveals LacZ expression in the trunk caudal to the otic vesicle and anterior to the presomitic mesoderm, with more intense staining in the lateral mesoderm (Fig. 4C). The anterior domain of staining extends rostral to the first somite but posterior to the otic vesicle. In the posterior region of the embryo, expression tapers off in the caudal somites and was absent in the presomitic mesoderm. Additional expression in the head was localized to the optic and forebrain vesicles. β-gal staining is also observed in the outflow tract of the heart (Fig. 4C′). In heterozygous *Hectd1^opm/+^* and homozygous *Hectd1^opm/opm^* mutants, the anterior and posterior boundaries of RARE activation were not altered, but staining overall was reduced compared with that in wild-type littermates (Fig. 4D,E). Staining in the forebrain, lateral mesoderm and the outflow tract of the heart tube were reduced in both heterozygous *Hectd1^opm/+^* and homozygous *Hectd1^opm/opm^* mutants compared with wild type (Fig. 4D,E). Together, these *in vitro* and *in vivo* assays demonstrate reduced retinoic acid signaling with mutation of *Hectd1* in both *Hectd1^opm/+^* and *Hectd1^opm/opm^* mutant embryos.

### Genetic interaction of *Hectd1* and retinoic acid synthesis alters PAA and aortic arch formation

**Fig. 5.**
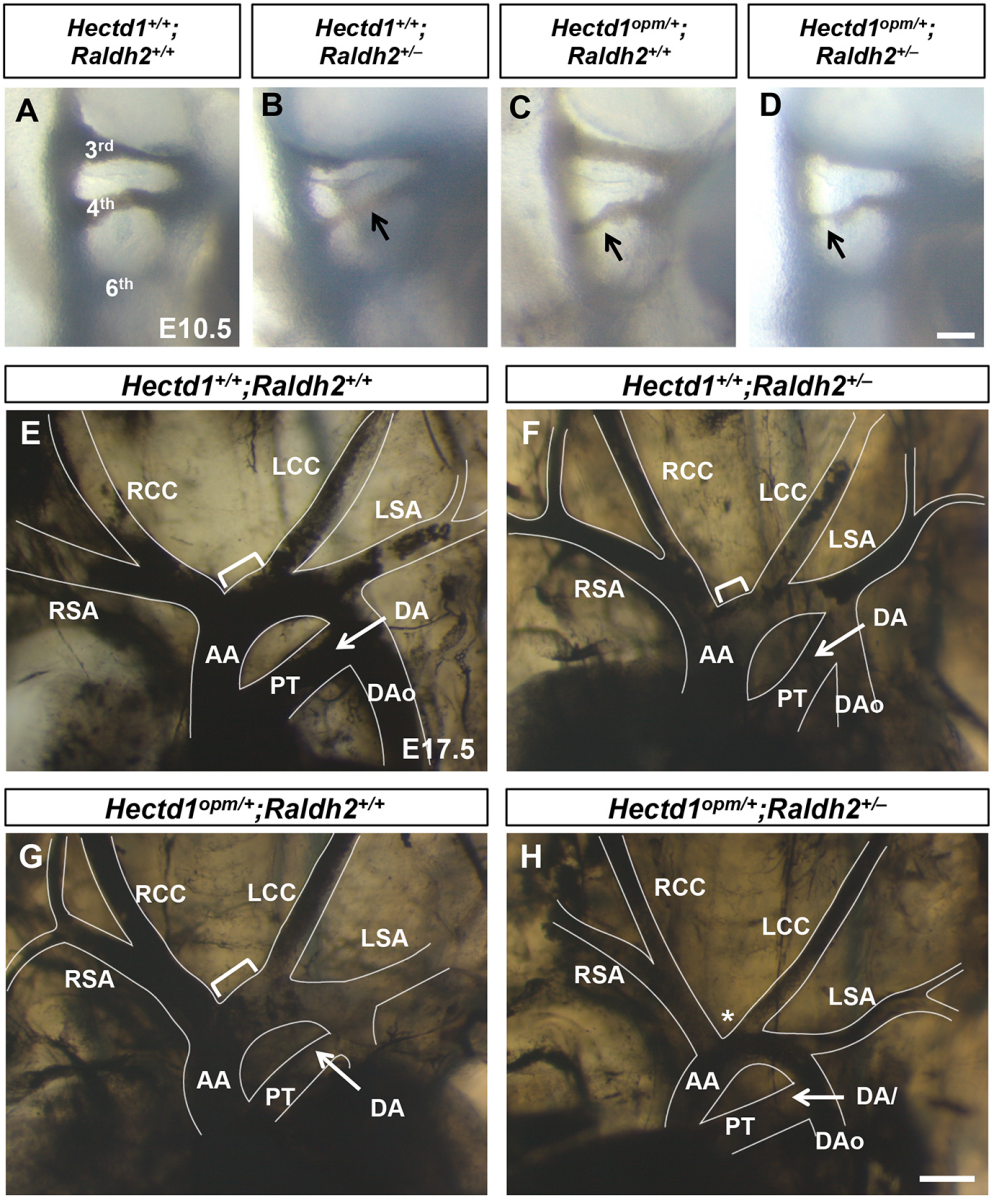
**Reduced *Raldh2* gene dosage results in PAA and aortic arch abnormalities in heterozygous *Hectd1^opm/+^* mutant embryos.** (A-H) Intracardiac ink injections of E10.5 (32- to 36-somite; A-D) and E17.5 (E-H) embryos to visualize the PAAs and aortic arch organization. (A-D) Right-sided views of E10.5 wild-type (*Hectd1^+/+^;Raldh2^+/+^*; A), *Hectd1^+/+^;Raldh2^+/−^* (B), *Hectd1^+/opm^;Raldh2^+/+^* (C) and *Hectd1^+/+^;Raldh2^+/+^* (D) embryos, showing the 3rd, 4th and 6th PAAs, with examples of hypoplastic 4th PAAs (arrows). Few wild-type (*n*=3/16, 19%), *Hectd1^+/+^;Raldh2^+/–^* (*n*=3/11, 27%) and *Hectd1^opm/+^;Raldh2^+/+^* (5/12, 42%) embryos showed hypoplastic 4th PAAs (Table 3). On the other hand, the 4th PAA was hypoplastic in two-thirds of double heterozygous *Hectd1^opm/+^;Raldh2^+/–^* embryos (*n*=11/16, 69%, *P*<0.0001, compared with wild type by Chi^2^ test). (E-H) At E17.5, 70% (7/10) of *Raldh2* heterozygotes (F) and 71% (5/7) *Hectd1* heterozygotes demonstrate similar length of the transverse arch between the brachiocephalic and left common carotid arteries (brackets) compared with wild type (E). This segment is shortened in *Hectd1^opm/+^;Raldh2^+/–^* double heterozygotes (H, asterisk), resulting in a shortened transverse arch between the brachiocephalic and left common carotid arteries in over half of embryos examined (4/7, 57%; *P*<0.05, Chi^2^ test). Scale bars: 50 µm (D) and 250 µm (H). AA, ascending aorta; DA, ductus arteriosus; DAo, descending aorta; LCC, left common carotid; LSA, left subclavian artery; PT, pulmonary trunk; RCC, right common carotid; RSA, right subclavian artery.

The intermediate increased expression of SHF markers and reduction in retinoic acid signaling in *Hectd1^opm/+^* heterozygous embryos raises the possibility that some heterozygotes might be more sensitive to alterations in retinoic acid exposure and more susceptible to aortic arch abnormalities. To test this, we challenged *Hectd1^opm/+^* heterozygotes with reduced retinoic acid exposure during embryogenesis and assayed the effects on PAA and aortic arch development. Retinoic acid exposure was decreased by reducing gene dosage of the retinoic acid synthesis enzyme *Raldh2* in *Hectd1^opm/+^;Raldh2^+/–^* double heterozygous embryos, which previous experiments demonstrate reduces activation of a RARE reporter by 20% in *Raldh2^+/–^* embryos (Maynard et al., 2013). Double heterozygous *Hectd1^opm/+^;Raldh2^+/–^* embryos were recovered at expected Mendelian ratios at weaning from *Hectd1^opm/+^* × *Raldh2^+/–^* crosses, indicating that lethal phenotypes were not significantly increased (*P*>0.05; Chi^2^ test; Table S1). To determine whether reduced retinoic acid synthesis results in PAA abnormalities, single and double heterozygous mutants were analyzed by intracardiac India ink injection at E10.5. Of the wild-type embryos observed, 19% (*n*=3/16) had hypoplastic left or right 4th PAAs, demonstrating a background phenotype in our mouse strain (Table 3). The percentage of embryos with hypoplastic 4th PAAs was higher, but not significantly different, in *Raldh2^+/–^* (3/11, 27%, *P*=0.60) or *Hectd1^opm/+^* (5/12, 42%, *P*=0.18) heterozygous embryos (Fig. 5A-C, Table 3). In contrast, the proportion of compound mutant embryos (*Hectd1^opm/+^;Raldh2^+/–^*) with affected PAAs was significantly increased compared with wild type (11/16, 69%, *P*<0.01; Fig. 5D, Table 3). The most common phenotypes observed were bilateral hypoplastic 4th PAAs (*n*=7), followed by hypoplasia of the right 4th PAA (*n*=3) and of the left 4th PAA (*n*=1, Fig. 5D, Table 3). These results indicate that combined reduction of *Hectd1* and retinoic acid synthesis interacts to cause hypoplasia of the 4th PAA, a less severe phenotype than the loss of 4th PAAs observed in homozygous *Hectd1^opm/opm^* mutants (Fig. 2, Table 2).

**Table 3.**
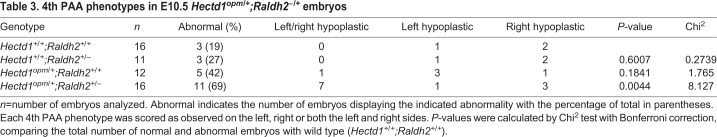
**4th PAA phenotypes in E10.5 *Hectd1^opm/+^;Raldh2^−/+^* embryos**

To determine whether the hypoplastic 4th PAA found in compound mutants results in abnormal aortic arch development, the aortic arch was visualized by intracardiac India ink injections in E17.5 embryos. The majority of wild-type (8/9; 89%), *Hectd1^+/+^;Raldh2^+/–^* (7/10; 70%) and *Hectd1^opm/+^;Raldh2^+/+^* (5/7; 71%) embryos displayed normal aortic arch architecture (Fig. 5E-G), with a statistically insignificant fraction exhibiting a shortened transverse arch between the brachiocephalic and left common carotid arteries (*P*>0.05; Chi^2^ test). This shortened transverse arch phenotype is less severe than the common origin of the brachiocephalic and left common carotid arteries observed in *Hectd1^opm/opm^* mutants (Fig. 1). Interestingly, the percentage of embryos with this benign aortic arch variant mirrors the percentage of embryos with hypoplastic 4th PAAs at E10.5. In contrast, over half of the double heterozygous (*Hectd1^opm/+^;Raldh2^+/–^*) embryos examined presented with this anatomical variation (4/7; 57%; *P*<0.05; Fig. 5H). This also reflects the proportion of double heterozygous embryos that presented with hypoplastic left 4th PAAs at E10.5.

## DISCUSSION

We demonstrate that *Hectd1* is required for normal aortic arch development through a previously undescribed interaction with retinoic acid signaling. Aortic arch abnormalities observed in *Hectd1^opm/opm^* mutants are similar to those described in mutant mouse models with reduced retinoic acid signaling, and include interrupted aortic arch, right-sided aortic arch as well as abnormalities of the subclavian and carotid arteries (El Robrini et al., 2016; Ghyselinck et al., 1997; Jiang et al., 2002; Kastner et al., 1994; Lee et al., 1997; Mendelsohn et al., 1994a; Niederreither et al., 2001; Pan and Baker, 2007; Stefanovic and Zaffran, 2017). In models with deficient retinoic acid signaling, aortic arch abnormalities arise from loss or hypoplasia of the left and right 4th PAAs that contribute to the aortic arch and right subclavian arteries, respectively (Hiruma et al., 2002; Li et al., 2012; Niederreither et al., 2003; Vermot et al., 2003). Similarly, our data indicate that the 4th PAA is typically bilaterally absent in the majority of *Hectd1^opm/opm^* mutant embryos, with a small proportion of embryos showing defects on only one side, consistent with the spectrum of aortic arch abnormalities observed. The endothelium of the PAAs is derived from the SHF, and reduced retinoic acid signaling causes altered patterning of the SHF, including increased expression of SHF markers such as *Nkx2.5*, *Isl1*, *Gata4* and *Fgf8* (Keegan et al., 2005; Li et al., 2012; Ryckebusch et al., 2008; Sirbu et al., 2008; Verzi et al., 2005; Wang et al., 2017). In *Hectd1^opm/opm^* mutants, expression of these markers is expanded. Thus, like retinoic acid signaling, *Hectd1* is required for normal SHF patterning, PAA development and remodeling into the mature aortic arch.

The 4th PAA phenotypes observed in E10.5 in *Hectd1^opm/opm^* mutant embryos are consistent with contribution of these arteries to the mature aortic arch and abnormalities observed at E14.5 (Hiruma et al., 2002; Stewart, 1964). For example, loss of the left 4th PAA is consistent with interrupted aortic arch type B and coarctation of the aortic arch, as the left 4th PAA contributes to the segment of the aortic arch between the left common carotid and subclavian arteries. On the other hand, loss of the right 4th PAA would affect the right subclavian artery, resulting in aberrant origin of the right subclavian artery and right-sided aortic arch phenotypes. As 90% of *Hectd1^opm/opm^* mutant embryos show bilateral loss of the 4th PAAs, there must be some compensatory mechanisms to result in this array of abnormalities of the mature artery network. Support for this idea comes from analysis of *Tbx1^+/–^;Raldh2^+/–^* double heterozygous embryos, where the majority of embryos show hypoplastic 4th PAAs at E10.5 but by E11.5 many of these recover, suggesting a delay in PAA formation (Ryckebusch et al., 2010). Interestingly, many *Hectd1* mutant embryos showed loss or hypoplasia of the 6th PAA, which is fated to form the ductus arteriosus. However, the ductus appeared to develop normally in *Hectd1^opm/opm^* homozygous and *Hectd1^opm/+^;Raldh2^+/–^* double heterozygous embryos. Thus, some compensation or delayed recovery likely rescues development of the ductus arteriosus in these embryos.

In contrast, linking abnormalities of the transverse arch, the segment of the aortic arch between the brachiocephalic and left common carotid arteries, to the 4th PAA defects is not as obvious. Several *Hectd1^opm/opm^* mutants observed had a common origin of the brachiocephalic and left common carotid arteries, a phenotype that is caused by a severe shortening of the transverse arch and is commonly given the misnomer ‘bovine arch’ (Moorehead et al., 2016; Shaw et al., 2003; Spacek and Veselka, 2012; Stewart, 1964). Interestingly, *Hectd1^opm/+^;Raldh2^+/–^* double heterozygotes also exhibited shortening of the transverse arch, possibly a less-severe variant of the common origin phenotype seen in *Hectd1^opm/opm^* mutants. Additionally at E10.5, *Hectd1^opm/opm^* mutants had a high frequency of missing left 4th PAAs, whereas the *Hectd1^opm/+^;Raldh2^+/–^* double heterozygotes had a high frequency of hypoplastic left 4th PAAs. Therefore, there might be a correlation between aplasia or hypoplastic left 4th PAAs and alterations in the length of the transverse arch. It is possible that these variant phenotypes arise due to alterations in the very extensive and complex remodeling of the symmetrical PAAs to the asymmetric aortic arch (Gupta et al., 2015, 2016; Hernandez et al., 2014). Therefore, although the left 4th PAA might not directly contribute to the transverse arch, its loss or underdevelopment can affect the length of the arch after the PAAs are remodeled. Although further study is needed to understand the origin of these phenotypes, our data suggest that decreased *Hectd1* gene dosage and/or retinoic acid signaling could be a novel contributor to this normal variant.

In light of the similarities in aortic arch abnormalities and underlying developmental mechanisms between *Hectd1^opm^* and retinoic acid pathway mutants, identification of RARA as a HECTD1-binding protein in a yeast two-hybrid screen provided an interesting potential molecular mechanism. The binding of fragments of HECTD1 and RARA observed in the yeast two-hybrid assay were confirmed in an *in vitro* binding assay, and binding of full-length overexpressed proteins was demonstrated in cells as well as binding of endogenous proteins in MEFs. Interestingly, the binding of endogenous proteins was detected under more stringent conditions than overexpressed full-length proteins, possibly suggesting cooperative binding involving a protein complex. This is further supported by the observation that expression of ligase-deficient HECTD1, similar to ligase-competent HECTD1, also enhanced ubiquitination of RARA. However, significant levels of ubiquitinated RARA are detected without HECTD1 transfection, likely due to expression of endogenous HECTD1, a prediction supported by the reduction of ubiquitinated RARA with small interfering RNA (siRNA)-mediated knockdown of HECTD1 in HEK cells and in *Hectd1^opm/opm^* mutant MEFs. Other ubiquitin ligases can influence the ubiquitinated state of RARA, including TRIM32, HACE1, RNF41 and RNF8 (Jing et al., 2008; Sato et al., 2012, 2011; Takano et al., 2004; Zhao et al., 2009). These or other uncharacterized ligases possibly cooperate with HECTD1 to influence RARA ubiquitination, stability and function.

The biological effects of retinoic acid signaling are mediated by retinoic acid receptors that bind to DNA as heterodimers consisting of RARs and retinoid X receptors (RXRs) each with A, B and G isoforms (Leid et al., 1992). These receptors act redundantly during development and heterodimers of RARs and RXRA are key mediators of retinoic acid signaling (Lee et al., 1997; Li et al., 2010). Our data demonstrate that HECTD1 binds RARA; however, deletion of RARA alone does not lead to significant defects in aortic arch development (Li et al., 1993; Lufkin et al., 1993). Similarly, RARB mutants do not show aortic arch defects (Luo et al., 1995; Mendelsohn et al., 1994a,b). Although mutation of RXRA results in defects in myocardial growth in the ventricle, aortic arch development appears normal (Sucov et al., 1994), Interestingly, HECTD1 binds RARA in a region of the receptor that is highly homologous to other RARs and RXRs. Based on this and the severe phenotype in *Hectd1^opm^* mutants, it is very likely that HECTD1 interacts with additional RARs during aortic arch development.

Our data do not address the molecular mechanism of how HECTD1 regulates retinoic acid signaling. HECTD1 has multiple substrates, a handful of which have been characterized (Aleidi et al., 2018; Duhamel et al., 2018; Li et al., 2015a, 2013; Sarkar and Zohn, 2013; Shen et al., 2017; Tran et al., 2013). One described mechanism of interest is the interaction of HECTD1 and the estrogen receptor (Li et al., 2015a). The estrogen receptor, like RARs, is activated by recruitment of transcriptional coactivators and corepressors that enhance or repress transcription, respectively (Perissi and Rosenfeld, 2005). HECTD1 can regulate the association of the estrogen receptor with coactivators and corepressors to modulate estrogen receptor signaling (Li et al., 2015a). Thus, it is possible that HECTD1 regulates retinoic acid signaling by a similar mechanism during aortic arch artery development.

The causes of variations in aortic arch organization are complex, with both monogenic and complex genetic etiologies with environmental contributions (Blue et al., 2012; Edwards and Gelb, 2016; Zaidi and Brueckner, 2017). Yet, how genes interact with environmental factors to cause these CHDs remains poorly understood. One challenge is the relatively few animal models and experimental paradigms available to investigate gene-environment interactions that cause abnormalities. Numerous genetic or environmental models of vitamin A deficiency or teratogenicity result in CHDs (Pan and Baker, 2007; Stefanovic and Zaffran, 2017), but very few studies address the role of susceptibility genes that interact with either vitamin A deficiency or subteratogenic exposures (Maynard et al., 2013). Our data demonstrate that *Hectd1^opm/+^* heterozygous mutant embryos show deficits in RARE activation as well as SHF patterning. This prompted us to evaluate whether heterozygous *Hectd1^opm/+^* mutant embryos would be more susceptible to defects by losing one copy of *Raldh2* and therefore reducing RARE activation by ∼20% (Maynard et al., 2013). Reduced *Raldh2* expression in *Hectd1^opm/+^* embryos resulted in a significant increase in the occurrence of hypoplastic 4th PAAs and subsequent shortening of the transverse arch between the brachiocephalic and left common carotid arteries. However, these phenotypes were not as severe as those observed in *Hectd1^opm/opm^* homozygous mutants. Therefore, it is possible that combined changes in *Hectd1* gene dosage and retinoic acid signaling result in a range of aortic arch abnormalities. Based on this work, we present a model system that can analyze how alterations in gene expression, environmental factors or both can alter aortic arch organization, furthering our understanding of genetic and environmental influences on heart development.

## MATERIALS AND METHODS

### Mouse strains and analysis of mutant phenotype

*Mus musculus* lines were maintained in the animal facility of Children's National Health System with all animal procedures in compliance with the National Institutes of Health and the Children's Research Institute Institutional Animal Care and Use Committee (IACUC) guidelines for animal use. *Hectd1^opm^*, *Hectd1^Gt(XC266)Byg^* (*Hectd1^XC^*), *Aldh1a2^tm1Gdu^* (*Raldh2^+/–^*) and the *Tg(RARE-Hspa1b/lacZ)12Jrt* (*RARE-LacZ*) reporter lines were maintained on the 129/SvJ background for upwards of ten generations and were previously described (Mic et al., 2002; Rossant et al., 1991; Zohn et al., 2007). Mice were fed standard mouse chow (Tekland, 8604) that contains 12.9 IU/g vitamin A as retinyl acetate. Female mice were between 3 months and 1 year old at the time of mating.

India ink injections were performed as described (Jianbin et al., 2008) using a 1:1 mixture of gelatin (Sigma-Aldrich) and India ink (Pelikan). For labeling of the aortic arch and arteries, freshly harvested E14.5 or E17.5 embryos were injected with the ink solution using an insulin needle followed by overnight fixation in 4% paraformaldehyde. For labeling of the PAAs, the ink solution was injected into the left ventricle of E10.5 embryos (32- to 36-somite stage) using a mouth pipette attached to a pulled glass needle. Aortic arch and artery abnormalities were diagnosed by three experienced observers. PAA phenotypes were scored blind to genotype by two experienced observers. Representative images were acquired using a Zeiss Lumar microscope with an Axiocam HRc camera (Zeiss) and Axiovision (4.6) software and processed using Adobe Photoshop (14.2). The frequency of each phenotype was calculated, and statistical significance between groups was determined by the Chi^2^ test with Bonferroni correction for multiple comparisons using GraphPad PRISM for Mac OS X (Version 6.0d).

For fluorescence imaging of the PAAs, embryos were fixed for 1 h in 4% paraformaldehyde and then dehydrated in a methanol series. Rehydrated embryos were incubated overnight at 4°C with anti-CD31/PECAM1 primary antibody (BD Pharmingen, 550274; 1:250), washed extensively, and then incubated overnight at 4°C with Alexa-Fluor-546-conjugated goat anti-rat IgG (H+L) cross-absorbed secondary antibody (Thermo Fisher Scientific, A-11081; 1:250). Embryos were dehydrated through a methanol series, cleared in benzyl alcohol:benzyl benzoate (BABB), and imaged in 1 μm optical sections using the Olympus Fluoview FV1000 confocal microscope and accompanying Olympus imaging software. Z-stacks were assembled to create 2D projections using Olympus, ImageJ (1.48v) and Fiji (2.0.0) software. Whole-mount *in situ* hybridization was performed as previously described (Holmes and Niswander, 2001; Zohn et al., 2006), with probes for *Fgf8* (Tanaka et al., 1992), *Nkx2.5* (Lints et al., 1993) and *Tbx1* (Chapman et al., 1996). Images were acquired using a Zeiss Lumar microscope with an Axiocam HRc camera (Zeiss) and Axiovision (4.6) software and processed using Adobe Photoshop (14.2). Whole-mount *in*-*situ*-stained embryos were sectioned and images taken on an Olympus BX63 upright microscope. Figures were assembled in Adobe Illustrator (17.1.0) and Microsoft PowerPoint for Mac 2011 (Version 12.6.2).

### qPCR

For qPCR, embryos were harvested at E9.5 and the pharyngeal region was dissected by bisecting the embryo between the maxillary and mandibular process of the first pharyngeal arch at the anterior end and between the heart tube and anterior limb bud at the posterior end (see Fig. 3D for dissection coordinates). The dissected pharyngeal region was dissociated in TRIzol (Invitrogen), RNA was extracted according to the manufacturer's instructions then diluted to 10 ng/μl. DNase digestion and complementary DNA (cDNA) synthesis were performed using the iScript gDNA Clear cDNA Synthesis Kit (Bio-Rad). RNA and cDNA concentrations were measured using a NanoDrop 1000 Spectrophotometer (Thermo Fisher Scientific). For qPCR, 2 μl cDNA (1000 ng/μl) was diluted in SsoAdvanced Universal SYBR Green Supermix (Bio-Rad). All gene-specific primers (10 nM, Table S2) were derived from previously published sequences and validated by melt-curve analysis (Lin et al., 2010; Maynard et al., 2013). qPCR was performed using the CFX96 Machine (Bio-Rad) under the following conditions: 95°C for 30 s, followed by 40 cycles of 95°C for 5 s and 60°C for 30 s. Expression levels were normalized to *Gapdh* and analyzed by CFX Manager Software (Bio-Rad, Version 3.1). Statistical significance of differences between the relative normalized expression of wild type and *Hectd1^opm/opm^* mutants for each marker was determined by unpaired two-tailed Student's *t*-test using GraphPad PRISM for Mac OS X (Version 6.0d). Outliers were tested using Grubb's outlier test on GraphPad PRISM (Version 6.0d) that identified a single *Hectd1^opm/opm^* mutant sample as an outlier and removed it from analysis.

### Biochemical analyses

pCMV-Myc-HECTD1(1-551), pCMV-HA-HECTD1 and pCMV-HA-HECTD1^C2579G^ (Hectd1^CG^; active site cysteine at position 2579 mutated to glycine) were previously described (Sarkar and Zohn, 2012). RARA-pcmv6 (Addgene, 35555) was modified with the addition of an amino terminal Myc-tag. Antibodies used in this study were anti-hemagglutinin (HA; Clontech, 631207; 1 µg/ml), anti-Myc (Clontech, 631206; 2 µg/ml), anti-RARA (Cell Signaling Technology, 2554; 1:1000), polyubiquitinylated conjugates monoclonal antibody (Clone FK1 Biomol, PW8805), FK2H (Biomol, PW0150; 1:1000), anti-HECTD1 (Novus Biologicals, H00025831; 1:500) and anti-GAPDH (Cell Signaling Technology, 2118; 1:1000). MEFs were prepared according to a standard protocol from individual E12.5 mouse embryo heads (Nagy et al., 2003), and HEK293T cells were from American Type Culture Collection (CRL-11268). Both cell lines were used at low passage numbers (<5). HEK293T cells were transfected using Lipofectamine 2000 Reagent (Invitrogen, 11668-019) according to the manufacturer's instructions. All biochemical data are representative of experiments performed at least twice.

Yeast two-hybrid and binding assays were performed as described (Sarkar and Zohn, 2012). Immunoprecipitation buffer (50 mM Tris-HCl, pH 7.5, 1 mM EDTA, 150 mM NaCl and 0.1% Triton X-100 with protease inhibitor cocktail) was used for less-stringent conditions, while RIPA buffer (Thermo Fisher Scientific, 89901) was used for stringent binding conditions. For ubiquitination assays, cells were treated for 4 h before lysis with 10 µM ALLN (Calbiochem, 208719). HEK293T cells were lysed 48 h post-transfection with a modified ubiquitination assay buffer (50 mM HEPES pH7.5, 5 mM EDTA, 150 mM NaCl, 1% Triton X-100, 10 mM N-ethylmaleimide, 1 mM PMSF, 2 µM ubiquitin aldehyde, 10 µM ALLN and 0.5% SDS) with the addition of a single complete protease inhibitor cocktail (Roche, 04693116001) and phosStop cocktail (Roche, 04906837001) tablet per 10 ml buffer.

### Analysis of RARE activation

Luciferase assays were performed on MEFs transfected using Lipofectamine LTX with Plus Reagent (Invitrogen, 15338-100) and with the βRE2-TK-Luc plasmid (Sucov et al., 1990) and pRL-TK plasmid (Promega, E2241). At 24 h post-transfection, cells were treated for 18 h with 10 µM ATRA, as indicated. Luciferase assays were performed using the Dual-Luciferase Reporter Assay System (Promega, E1910) and a Turner Biosystems luminometer (Model 2030-000). Statistical significance of RARE activation between wild-type and *Hectd1^opm/opm^* MEFs was determined by the unpaired two-tailed Student's *t*-test using GraphPad PRISM for Mac OS X (Version 6.0d). For quantification of β-gal expression in E9.5 *RARE-LacZ* embryos, whole-embryo littermate pairs were dissociated in 1× Reporter Lysis Buffer from the Beta-Galactosidase Enzyme Assay System (Promega) as described (Maynard et al., 2013). The level of absorbance at 420 nm of samples was interpolated to a standard β-gal curve to determine activity in milliunits using GraphPad PRISM for Mac OS X (Version 6.0d). Protein concentration was determined using a BCA Protein Quantification Kit (Abcam). The final measure of RARE activation was calculated by dividing milliunits of β-gal activity by protein concentration of each embryo using Microsoft Excel for Mac 2011 (Version 12.6.2). β-gal and protein quantification were determined using a Fluostar Optima Plate Reader (BMG Labtech). Statistical significance between wild-type and *Hectd1^opm/+^* or *Hectd1^opm/opm^* values was determined by the unpaired two-tailed Student's *t*-test using GraphPad PRISM for Mac OS X (Version 6.0d). Whole-mount β-gal staining was performed as described (Nagy et al., 2003). Images were acquired using a Zeiss Lumar microscope with an Axiocam HRc camera (Zeiss) and Axiovision (4.6) software and processed using Adobe Photoshop (14.2).

## Supplementary Material

Supplementary information
