# Supplementary material for: The ubiquitin ligase HECTD1 promotes retinoic acid signaling required for development of the aortic arch
Source: Dis Model Mech. 2019 Jan 11;12(1):dmm036491. doi: 10.1242/dmm.036491 (PMC6361158; doi:10.1242/dmm.036491)
Supplement: Supplementary information [file dmm-12-036491-s1.pdf]

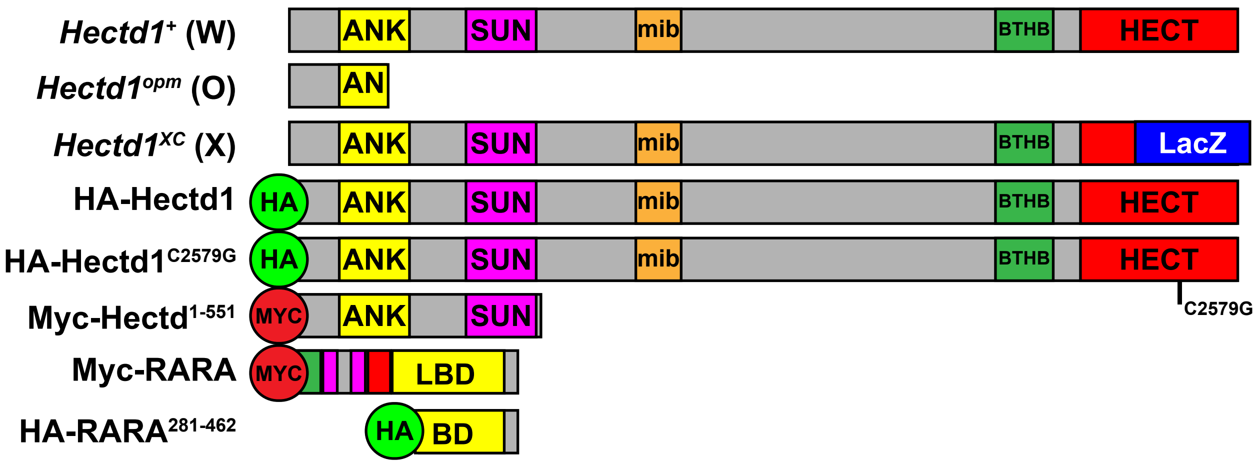

Figure S1. Schematic diagram of the mice and plasmid constructs used in this study. Numbers refer to the amino acid residues. Hectd1 contains ankyrin (ANK), Sad1/UNC (SUN), Mindbomb (MIB), Basic Tilted Helix Bundle (*BTHB*) and Homologous to the E6-AP Carboxyl Terminus (HECT) *domains*. An N-terminal fragment of HECTD1 encompassing the ANK domain, HECTD1(1-551), was used as bait to screen an E11.5 mouse cDNA library. Three overlapping clones encompassing amino acids 281 to 462 of the retinoic acid receptor alpha (RARA) were identified. This fragment contains the Ligand Binding Domain (LBD) and is highly conserved in all retinoic acid and retinoid X receptors.

Table S1. Mendelian ratios of expected genotypes were obtained from *Hectd1*$^{opm/+}$ X *Raldh2*$^{+/–}$ crosses

	Hectd1$^{+/+}$; *Raldh2*$^{+/+}$ (%)	*Hectd1*$^{+/+}$; *Raldh2*$^{+/–}$ (%)	*Hectd1*$^{opm/+}$; *Raldh2*$^{+/+}$ (%)	*Hectd1*$^{opm/+}$; *Raldh2*$^{+/–}$ (%)	p-value	Chi^2
**Observed**	13 (18)	23 (32)	21 (29)	15 (21)		
**Expected**	18 (25)	18 (25)	18 (25)	18 (25)	0.5892	1.920

p-values were calculated by the Chi^2 test comparing the numbers of observed and expected pups

Table S2. Primer Sequences for qPCR Assay

Marker	Primer Sequence	Reference
Isl1	**F** 5' CACTATTTGCCACCTAGCCAC<br>**R** 5' AAATACTGATTACACTCCGCAC	(Lin et al., 2010)
Gata4	**F** 5' TCTCACTATGGGCACAGCAG<br>**R** 5' ACAGCACTGGATGGATGGAG	(Lin et al., 2010)
Mef2c	**F** 5' GTCAGTTGGGAGCTTGCACTA<br>**R** 5' CGGTCTCTAGGAGGAGAAACA	(Lin et al., 2010)
Tbx1	**F** 5' CGAGATGATCGTCACCAAGG<br>**R** 5' CCAGGAGGAGCTATGGAAAG	(Maynard et al., 2013)
Fgf8	**F** 5' TGGAAGCAGAGTCCGAGTTC<br>**R** 5' TGTGAATACGCAGTCCTTGC	(Maynard et al., 2013)
GAPDH	**F** 5' CTGACGTGCCGCCTGGAGAAA<br>**R** 5' GTTGGGGGCCGAGTTGGGATAGG	(Maynard et al., 2013)
